# Best fit framework synthesis of qualitative studies on factors associated with medication nonadherence in people with type 2 diabetes using the COM‐B model

**DOI:** 10.1002/bcp.70059

**Published:** 2025-04-02

**Authors:** Vivien Teo, John Weinman, Kai Zhen Yap

**Affiliations:** ^1^ Institute of Pharmaceutical Sciences, King's College London London UK; ^2^ Department of Pharmacy National University of Singapore Singapore

**Keywords:** diabetesmellitus, type 2, medication adherence, motivation, qualitative research

## Abstract

This review aimed to synthesize factors associated with medication nonadherence among people with type 2 diabetes (PwT2D), using the Capability, Opportunity, Motivation and Behaviour (COM‐B) model as the a priori model.

Studies published between January 2014 and April 2024 were searched on five databases. Studies were included if they recruited PwT2D aged >18 years, investigated factors associated with adherence to oral and/or nonoral medications for diabetes, used qualitative research methods, were conducted in a community setting, were in English language and had accessible full‐text articles. Best fit framework synthesis was undertaken, which led to the development of a hypothesized COM‐B variant model specific to medication nonadherence among PwT2D. Study quality was assessed using published criteria to evaluate whether the study was adequately reported.

Twenty‐two studies were included. Factors were mapped onto the COM‐B model: physical capability (e.g., difficulty injecting insulin independently), psychological capability (e.g., understanding about diabetes), physical opportunity (e.g., cost of medication), social opportunity (e.g., quality of communication and relationship with healthcare providers), automatic motivation (e.g., habit formation) and reflective motivation (e.g., perceived necessity and effectiveness of medications). Reflective motivation had the most themes, while physical capability only had one theme. Personality was a theme that could not be mapped onto the model. Interactions between some COM‐B components (e.g., capability and motivation) were observed.

This theoretically grounded synthesis may facilitate future intervention development by formulating a programme theory and identifying behaviour change techniques to address the identified factors.

## 1 INTRODUCTION

### 1.1 Background

Diabetes is a major public health burden. According to the International Diabetes Federation Report in 2021, 10.5% of the adults worldwide have diabetes and the prevalence was projected to increase to 12.2% by 2045.^1^, ^2^ More than 90% of the people with diabetes have type 2 diabetes mellitus (T2DM)^2^ and this prevalence is likely to rise with urbanization and ageing population.^2^, ^3^

The global healthcare cost of diabetes rose from US$232 billion in 2007 to US$966 billion in 2021 and is expected to hit US$1.05 trillion by 2045.^1^ In 2021, diabetes and its complications accounted for 12.2% of all adult deaths globally.^1^

Medications are the cornerstone of diabetes management, yet medication nonadherence has been observed in at least 50% of the people with type 2 diabetes (PwT2D).^4^, ^5^ Medication nonadherence results in poor glycaemic control^6^ and is an independent risk factor for all‐cause mortality among PwT2D.^7^

Many questionnaires, such as the Beliefs about Medicines Questionnaires^8^ investigate people's views about their medications. However, there remains significant variance in medication adherence that cannot be explained^9^ by these quantitative measures, which are primarily for testing specific hypotheses.^10^ Qualitative studies are necessary for exploring the reasons underlying people's behaviour in greater depth, revealing new insights that may explain their medication‐taking behaviour^10^ before conducting focused hypothesis testing.

An earlier qualitative meta‐synthesis elucidated different barriers of medication adherence among PwT2D.^11^ Without using a theoretical model,^11^ there was little information on how these factors could be targeted to improve medication adherence.^12^ Theory is important for identifying constructs that influence medication adherence and for selecting intervention components to target these.^13^ The lack of a theoretical basis for designing interventions^14^ may explain the suboptimal outcomes of current interventions.^15^

**FIGURE 1:**
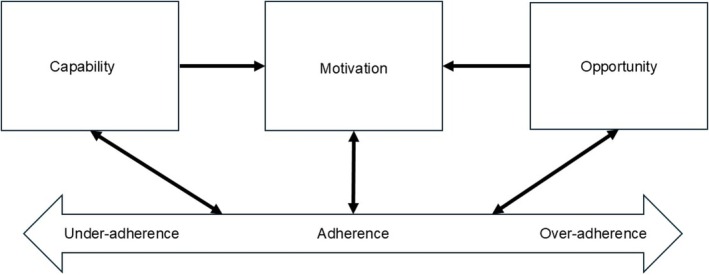
Application of the Capability, Opportunity, Motivation and Behaviour model to medication adherence.^12^

Unlike earlier theories that focus on specific behavioural aspects,^12^ the Capability, Opportunity, Motivation and Behaviour (COM‐B) model is a comprehensive, overarching behavioural model developed by evaluating and integrating various behaviour change intervention frameworks.^16^, ^17^ This model posits that capability, opportunity and motivation are necessary components to perform a behaviour, which can in turn affect these components (Figure 1).^12^, ^17^ Capability can be physical or psychological, opportunity can be physical or social, while motivation can be reflective or automatic (Table 1). The COM‐B model is the core of the behaviour change wheel, a theoretically derived method that facilitates the selection of behaviour change techniques targeting specific COM‐B components in intervention design.^17^

**TABLE 1:** Definition of the COM‐B model.

COM‐B component	Definition
Capability	
Physical	Capacity to engage in necessary physical processes, having the physical skills, strength and stamina^12^, ^17^
Psychological	Capacity to engage in necessary thought processes, having the cognitive functioning, memory, knowledge and understanding of T2DM and treatment^12^, ^16^, ^17^
Opportunity	
Physical	Physical opportunity in the environment, including locations, resources, costs, accessibility, availability, characteristics of medication and treatment regimen^12^, ^16^
Social	Culture, social norms, interpersonal influence^12^, ^16^
Motivation	
Reflective	Conscious planning, evaluation, belief, perception about T2DM and treatment, self‐efficacy^12^, ^16^, ^17^
Automatic	Emotion, feeling, mood, urge, reflex that emerge from associative learning or inherent tendencies, routines and habits^16^, ^17^

Abbreviations: COM‐B: Capability, Opportunity, Motivation and Behaviour model; T2DM: type 2 diabetes mellitus.

### 1.2 Aim

Our review aimed to synthesize qualitative studies on factors associated with medication nonadherence among PwT2D, using the COM‐B model^12^, ^16^, ^17^ as the a priori model.

## 2 METHODOLOGY

All studies published in January 2014–January 2022 were screened, extracted, analysed and appraised by two reviewers (VT and CJ) independently, before they were compared and reconciled. Any discrepancy was resolved through discussion with a third reviewer (KZY/JW) if required. Studies published in February 2022–April 2024 that were found in later searches using the same search strategy were screened, extracted, analysed and appraised by VT. The themes were refined by VT and discussed with KZY and JW.

This review was registered with PROSPERO (CRD42023434654) and followed The Preferred Reporting Items for Systematic Reviews and Meta‐analysis (PRISMA) statement.^18^

### 2.1 Literature search

**FIGURE 2:**
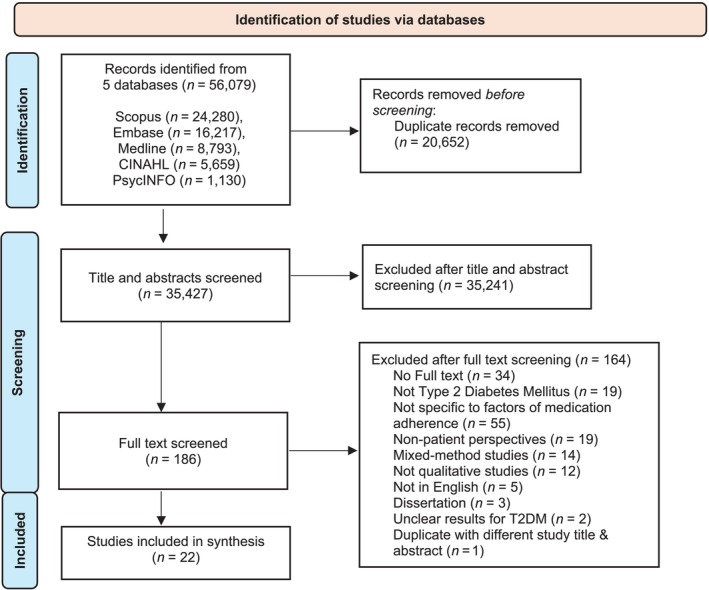
Flow diagram of studies included.

Figure 2 summarizes the literature search process. Following the search period of an earlier synthesis that ended in 2013,^11^ studies published from 1 January 2014 to 16 April 2024 were searched on Medline, Embase, CINAHL, PsychINFO and Scopus. The search terms focused on diabetes and medication adherence. The term *adherence* was used, as it reflects the mutual agreement of PwT2D and healthcare providers (HCPs) on the treatment in patient‐centred care^19^ and is commonly used in studies following the World Health Organization's definition in 2003.^20^

The search strategy is shown in Appendix 1. Endnote 20 was used.

### 2.2 Eligibility criteria

Studies were included if they recruited PwT2D aged >18 years, investigated factors associated with adherence to oral and/or nonoral medications for diabetes, used qualitative research methods, were conducted in a community setting, were in English‐language and had accessible full‐text articles. Studies were excluded if they used quantitative or mixed methods, included non‐patient views, were not specific to factors associated with adherence to diabetes medications, presented unclear results for PwT2D as other clinical groups were also recruited, were dissertation or conference papers.

### 2.3 Extraction and synthesis

Details on the study population, methodology, authors' findings and participants' verbatim quotations were extracted. Only data from the Results section were analysed.

Best fit framework synthesis (BFFS)^21^ was undertaken. Data extracted from the studies were coded into themes and mapped onto the COM‐B model deductively^21^ based on definitions from earlier studies.^12^, ^16^, ^17^ To allow for data that did not fit into the COM‐B model, themes outside of the COM‐B model were generated inductively through thematic analysis, reviewers' interpretation and constant comparison of themes across studies.^21^ This led to the development of a hypothesized COM‐B variant model aiming to include themes aligned with the COM‐B model and themes outside the COM‐B model. The themes were categorized into facilitators and barriers to medication adherence. Interactions between the COM‐B components were hypothesized based on the included studies. Nvivo 14 was used for analysing data.

### 2.4 Study quality appraisal

Our review followed BFFS authors' recommendation of using published criteria that appraised whether the question and study design, participant selection, data collection and analysis method were adequately reported.^21^, ^22^ A study was regarded as *adequately reported* if details on at least two out of four criteria were provided.^22^ These criteria assessed explicit description of a study, reduced reviewers' subjective judgements on the theoretical perspectives, validity and reliability of these studies.^23^

### 2.5 Comparison with the a priori model, dissonance and sensitivity

The resultant hypothesized model was compared with the COM‐B model. To test the validity of this review, the presence of dissonance in the form of contradictory views was assessed.^21^ To evaluate the impact of reporting quality, sensitivity analysis was considered to see if the synthesis was affected by excluding inadequately reported studies.^21^

## 3 RESULTS

### 3.1 Study characteristics

Twenty‐two studies consisting of 434 PwT2D were included in our review. Eighteen studies conducted semi‐structured or in‐depth interviews; four studies conducted focus groups. Fifteen studies reported the mean age of the PwT2D, ranging 51.7–70 years old.^24^, ^25^ Eight studies incorporated theories into their study designs, including the Theory of Planned Behaviour,^26^, ^27^ Necessity‐Concerns Framework,^28^, ^29^ Roy adaptation model^24^, ^25^ and Socioecological Model.^30^, ^31^ The studies were undertaken in various countries, for example the USA (*n =* 6),^24^, ^25^, ^30^, ^32^, ^33^, ^34^ Australia (*n =* 3),^35^, ^36^, ^37^ Singapore (*n =* 2)^38^, ^39^ and UK (*n =* 1).^28^ Twelve studies included PwT2D on both oral and injectable medications. Ten studies focused on PwT2D on injectable medication only, oral medication only or did not specify the medication type. Only two studies specified the exact oral medications, including metformin^26^, ^29^ and sulfonylureas.^26^, ^29^

All studies described their research question and study design, participant selection, data collection, and analysis method adequately. Therefore, sensitivity analysis was not conducted. Dissonance in the form of contradictory findings was readily identifiable.

Table 2 summarizes the study characteristics, with more details in Appendix 2.

**TABLE 2:** Study characteristics.

Study	Methodology	Participants	Country	Quality assessment
Jannuzzi *et al*. 2014^26^	Semi‐structured interview; Theory of planned behaviour	17 PwT2D, mean age 59.8	Brazil	Adequately described
Widayanti *et al*. 2021^40^	Semi‐structured interview; Conceptual model of Patients' Lived Experiences with Medicines (PLEM)	51 PwT2D, unspecified mean age	Indonesia	Adequately described
Baghikar *et al*. 2019^30^	Semi‐structured interview; Social Ecological Model	27 PwT2D, mean age 57	USA	Adequately described
Hassali *et al*. 2014^41^	Semi‐structured interview; Phenomenological approach	13 PwT2D, mean age 59.8	Malaysia	Adequately described
Patel *et al*. 2015^28^	Semi‐structured interview; Necessity‐concerns framework	18 PwT2D, unspecified mean age	UK	Adequately described
Liu *et al*. 2022^38^	Semi‐structured interview	21 PwT2D, mean age 61	Singapore	Adequately described
Mathew *et al*. 2022^39^	Semistructured interview	21 PwT2D, median age 63	Singapore	Adequately described
Bockwoldt *et al*. 2017^25^	Semi‐structured interview, Roy adaptation model	15 PwT2D, mean age 51.7	USA	Adequately described
Alhaddad *et al*. 2015^42^	Semi‐structured interview	20 PwT2D, mean age 53.7	Kuwait	Adequately described
Rezaei *et al*. 2019^43^	Semi‐structured interview	12 PwT2D, mean age 52	Iran	Adequately described
Ahmad *et al*. 2021^35^	Semi‐structured interview	23 PwT2D, median age 39	Australia	Adequately described
Onwuchuluba *et al*. 2021^31^	Semi‐structured interview; Socioecological framework	25 PwT2D, unspecified mean age	Nigeria	Adequately described
Sapkota *et al*. 2016^36^	Semi‐structured interview	48 PwT2D, median age 55.5	Australia and Nepal	Adequately described
Jiraporncharoen *et al*. 2020^44^	Semi‐structured interview; World Health Organization framework for medication adherence	24 PwT2D, mean age 62	Thailand	Adequately described
Polonsky *et al*. 2021^32^	Semi‐structured interview	36 PwT2D, mean age 58.3	USA	Adequately described
Alzubaidi *et al*. 2015^37^	Semi‐structured individual interview and group interview	100 PwT2D, mean age 57–60 for different groups	Australia	Adequately described
Okazaki *et al*. 2022^45^	Qualitative phone interview	6 PwT2D, mean age 60.5	Japan	Adequately described
Habte *et al*. 2017^29^	In‐depth interview; Necessity‐concerns model	39 PwT2D, unspecified mean age	Central Ethiopia	Adequately described
Guenette *et al*. 2015^27^	Focus group; Theory of planned behaviour	45 PwT2D, mean age 63.8	Canada	Adequately described
Shiyanbola *et al*. 2018^33^	Focus group; Phenomenology approach	40 PwT2D, mean age 53	USA	Adequately described
Bockwoldt *et al*. 2016^24^	Focus group;Roy adaptation model	13 PwT2D, mean age 52 for mid‐life group, 70 for older group	USA	Adequately described
Hsu *et al*. 2014^34^	Focus group	45 PwT2D, mean age 69.3	USA	Adequately described

Abbreviation: PwT2D: people with type 2 diabetes; UK, United Kingdom; USA, United States of America.

### 3.2 Hypothesised COM‐B variant model

**FIGURE 3:**
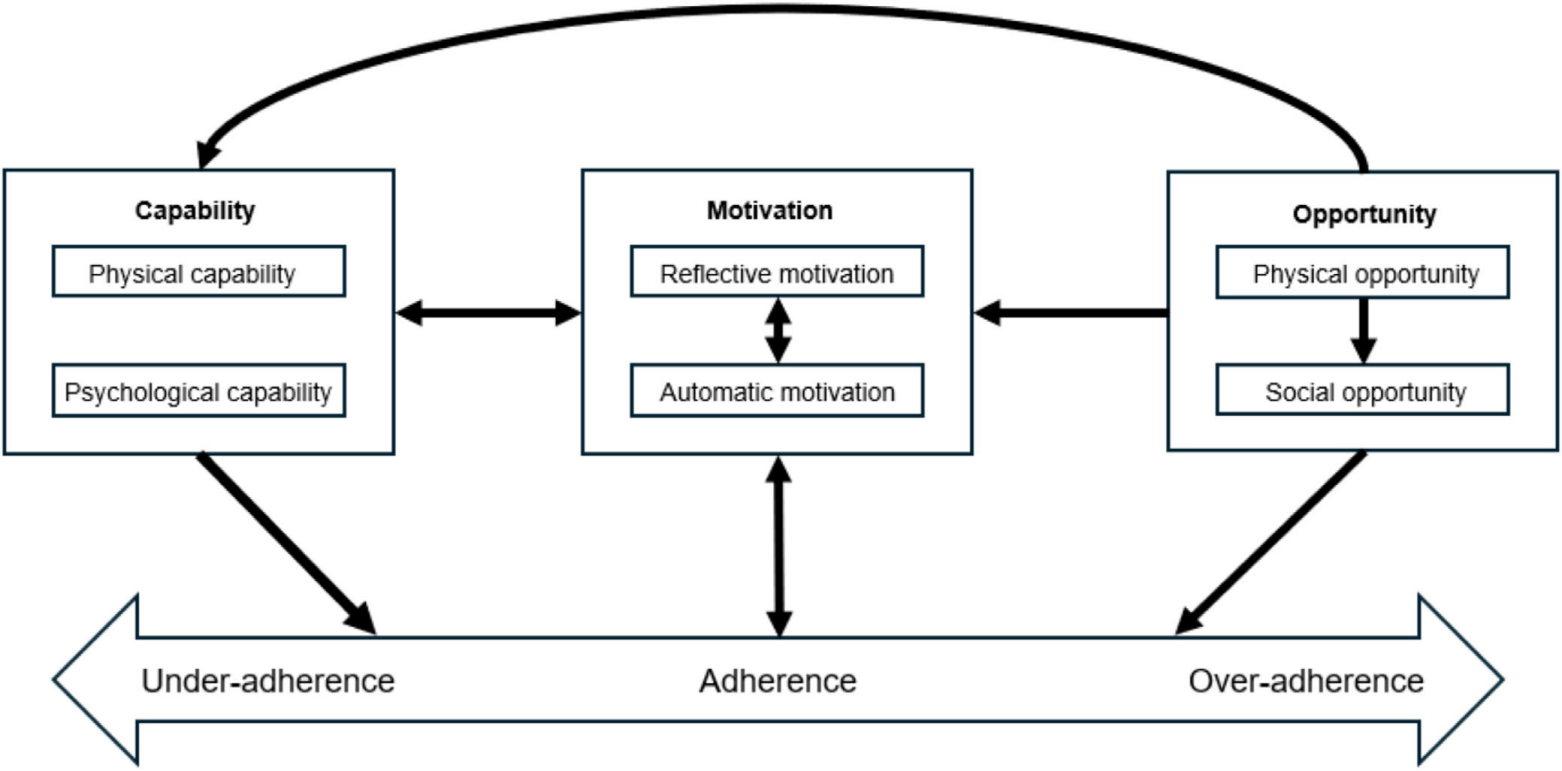
Hypothesized Capability, Opportunity, Motivation and Behaviour variant model for medication adherence among people with type 2 diabetes.

Table 3 lists themes that could and could not be mapped onto the COM‐B model, along with the numbers of the studies supporting each theme. Reflective motivation had the most themes, while only one theme was related to physical capability. Personality was a new theme that could not be mapped onto the model. Figure 3 presents a COM‐B variant model hypothesized from the findings of this review.

**TABLE 3:** Themes that could and could not be mapped onto the COM‐B model and the number of studies supporting each theme (n).

Facilitator	*n*	Barrier	*n*
Physical capability			
‐	‐	Difficulty injecting insulin independently	4
Psychological capability			
Good understanding about medication	9	Lack understanding about medication	9
Good understanding about diabetes	3	Lack understanding about diabetes	9
		Forgetfulness	9
		Confusion about diabetes effect and medication side effects	2
Physical opportunity			
Affordable medication and consumable cost	4	High medication and consumable cost	10
Good availability and access to medication and consumable	2	Lack availability and difficult access to medication and consumable	4
Satisfactory clinic and pharmacy service	1	Unsatisfactory clinic and pharmacy service	4
Easy access to HCPs	3	Difficult access to HCPs	3
		Complex and stricter regimens	6
		Storage requirements of medication	3
		Medication was very big for swallowing	1
Social opportunity			
Good communication and relationship with HCP	16	Lack and poor communication and relationship with HCP	14
Other people's experience with medication	1	Other people's negative experience with medication	6
Other people's negative experience with diabetes	3	Other people's experience living with diabetes without medication	1
Presence of family support	9	Absence of family support	2
Positive peer support	1	Negative peer influence	1
		Stigma surrounding diabetes, insulin, injection, medication taking in public	14
		Fear of burdening or worrying loved ones	4
Automatic motivation			
Successful habit and routine formation	9	Unsuccessful habit and routine formation	3
		Fear of needle, injection, blood	11
		Negative feelings towards medications	7
		Burnout and self‐care fatigue	4
		Realized injection is not painful after trying	1
		Repelled to inject body parts apart from fingers	1
Reflective motivation			
Believe in the necessity and effectiveness of medications	17	Disbelieve in the necessity and effectiveness of medications	12
Presence of personal ownership	16	Absence of personal ownership	9
Positive perception of certain medications and brands	2	Negative perception of certain medications and brands	2
Concerns about diabetes symptoms and complications	15	Experience of diabetes symptoms and complications despite medication adherence	2
Prioritization of diabetes due to other medical condition	1	De‐prioritization of diabetes due to other medical conditions	1
Hope to avoid or stop insulin in the future	8	Concerns about side effects of medications	19
Self‐efficacy	6	Greater value placed on competing priorities	11
		Self‐adjustment of medication based on food, lifestyle, glucose levels, side effects and symptoms	11
		Self‐identity and concept of being sick	9
		Negative association of insulin with severe diabetes, seeing insulin as the last resort	7
		Fatalism	7
		Preference for alternative medications	6
		Preference for nonpharmacological management over medications	4
		Concern about needle hygiene, device issues and source of insulin	3
Theme that could not be mapped onto the COM‐B			
‐		Personality	1

Abbreviation: HCP: Healthcare providers.

#### 3.2.1 Capability

One physical and four psychological capability factors were identified.

##### Physical capability

Difficulty injecting insulin independently was the only theme coded onto physical capability.^36^, ^40^, ^41^, ^43^ This barrier may be secondary to loss of ability from old age,^43^ visual and coordination challenges.^41^

##### Psychological capability

Forgetfulness,^25^, ^26^, ^27^, ^31^, ^33^, ^35^, ^38^, ^40^, ^41^ understanding of diabetes^28^, ^29^, ^31^, ^33^, ^35^, ^36^, ^37^, ^42^, ^43^, ^45^ and medications,^25^, ^26^, ^28^, ^29^, ^31^, ^32^, ^34^, ^35^, ^37^, ^38^, ^39^, ^41^, ^42^, ^43^, ^45^ confusion about diabetes effects and medication side effects,^29^, ^44^ emerged as themes from the studies.

Forgetfulness was often mentioned.^25^, ^26^, ^27^, ^31^, ^33^, ^35^, ^38^, ^40^, ^41^ PwT2D forgot their medications, for example at mealtime^27^ or when they were home late.^38^

Some PwT2Ds' lack of understanding about diabetes also influenced their medication adherence.^28^, ^29^, ^31^, ^33^, ^35^, ^37^, ^42^, ^43^, ^45^ They were unaware of the severity of diabetes^35^, ^37^ and thought “diabetes was like the flu” that they would recover from soon.^42^ Conversely, other PwT2Ds' good understanding about diabetes^28^, ^35^, ^36^ and medications^25^, ^26^, ^28^, ^31^, ^32^, ^35^, ^37^, ^38^, ^45^ facilitated adherence. These factors may well overlap with some of the illness perception factors, which are described in Section 3.2.5 on the interactions between COM‐B components.

PwT2D could be confused about the effects of diabetes and side effects of medications^29^, ^44^ owing to limited understanding about diabetes and medications. Some PwT2D attributed diabetes complications, such as eye damage to medications and side effects of medications, such as hypoglycaemia to diabetes.^29^

#### 3.2.2 Opportunity

Both physical and social opportunity factors were noted in the studies.

##### Physical opportunity

Physical opportunity factors included cost,^25^, ^26^, ^30^, ^31^, ^32^, ^34^, ^36^, ^38^, ^40^, ^41^, ^43^ availability and access to medications and consumables,^29^, ^32^, ^36^, ^40^, ^42^ clinic and pharmacy service quality,^31^, ^34^, ^40^, ^42^ access to HCPs,^27^ ^,^ ^32^ ^,^ ^34^ ^,^ ^39^ ^,^ ^40^ ^,^ ^42^ complex and stricter regimens,^28^, ^29^, ^36^, ^40^, ^41^, ^43^ medication size^31^ and storage requirements.^29^, ^36^, ^40^

Many PwT2D were concerned about the cost of medications and consumables, which impeded their medication adherence.^25^, ^30^, ^31^, ^32^, ^34^, ^36^, ^38^, ^40^, ^41^, ^43^ Some PwT2D did not take their medications because they could not afford them^30^, ^31^, ^32^ and had to rely on others to cover their medication expenses.^43^ Affordable medications and consumables^26^, ^32^, ^36^, ^38^supported by subsidies^36^, ^38^ for example, facilitate medication adherence.

Four studies also described a lack of availability and access to medication.^29^, ^36^, ^40^, ^42^ Some PwT2D had to travel long distances to get them^40^ or even use expired medications.^29^ Difficult access to HCPs discouraged medication adherence,^27^, ^40^, ^42^ while easy access to HCP encouraged medication adherence,^32^, ^34^, ^39^ for example when HCPs proactively followed up with PwT2D to address their medication concerns.^32^, ^39^

Medication adherence was supported when clinics and pharmacy provided good services, such as convenient medication refill services via a phone or automatic system.^34^ Conversely, PwT2D were reluctant to see their HCPs and obtain their medications if clinic and pharmacy services were not satisfactory,^31^, ^34^, ^40^, ^42^ such as overcrowded clinics^31^ and long wait times.^40^, ^42^

Some PwT2D felt that insulin required *stricter adherence* ^29^ and expressed difficulty adhering to the requirements of taking their meals regularly,^28^, ^40^ timing their meals and medications.^28^, ^29^, ^40^ Multiple dosing frequency^26^, ^32^, ^35^, ^44^ and the need to take many medications^29^, ^33^, ^42^, ^44^ were some of the barriers faced. Moreover, the refrigeration requirements of insulin caused much inconvenience in transporting and storing insulin,^29^, ^36^, ^40^ especially for PwT2D who did not have a refrigerator.^31^

##### Social opportunity

Communication and relationship with HCPs,^24^, ^26^, ^27^, ^28^, ^29^, ^30^, ^31^, ^32^, ^33^, ^34^, ^35^, ^36^, ^37^, ^38^, ^39^, ^41^, ^42^, ^43^, ^44^, ^45^ other people's experience with diabetes^26^, ^28^, ^38^, ^44^ and medications,^25^, ^28^, ^33^, ^37^, ^41^, ^44^ family support,^24^, ^25^, ^26^, ^27^, ^30^, ^31^, ^35^, ^37^, ^42^, ^44^ peer influence,^26^, ^27^ stigma surrounding diabetes and medication use,^24^, ^25^, ^27^, ^28^, ^29^, ^31^, ^35^, ^36^, ^37^, ^38^, ^41^, ^42^, ^43^, ^45^ and the fear of burdening or worrying loved ones^24^, ^27^, ^35^, ^36^ were the social opportunity factors highlighted in the studies.

The quality of communication and relationship with HCPs were often mentioned as factors influencing medication adherence. There was a lack of communication and poor relationship with HCPs.^24^, ^27^, ^29^, ^30^, ^31^, ^33^, ^35^, ^37^, ^38^, ^39^, ^42^, ^43^, ^44^, ^45^ PwT2D were told that they “deserved injections”,^29^ and they felt blamed by HCPs for their poor diabetes control.^24^, ^29^, ^39^ HCPs were described as being paternalistic,^42^ reprimanding,^39^ and threatening towards PwT2D.^42^ Hence, PwT2D refused to discuss their health issues with HCPs,^37^, ^39^ reflecting a lack of trust in HCPs.^27^, ^30^, ^31^, ^43^, ^44^, ^45^ They preferred talking to people other than their HCPs, such as their family about their medications,^37^, ^43^ which sometimes resulted in nonadherence.^37^ Good communications and relationships with HCPs are key to medication adherence.^25^, ^26^, ^28^, ^30^, ^32^, ^33^, ^34^, ^35^, ^36^, ^37^, ^38^, ^39^, ^41^, ^42^, ^44^, ^45^ PwT2D's fear and concerns about medications were addressed.^28^, ^30^, ^35^, ^37^, ^39^, ^45^ They felt “approved”^26^ and being cared for.^28^, ^39^ This trust in HCPs is essential,^26^, ^28^, ^30^, ^33^, ^35^, ^36^, ^37^, ^38^, ^39^, ^41^, ^44^, ^45^ and when they trusted their HCP, they trusted the medications prescribed.^36^

The absence of family support hampered medication adherence,^31^, ^44^ while its presence promoted medication adherence.^25^, ^26^, ^27^, ^30^, ^31^, ^35^, ^37^, ^42^, ^44^ Families provided emotional and practical support,^31^, ^42^ for example by reminding PwT2D to take their medications^25^, ^26^, ^35^, ^42^ and organizing their medications in containers.^26^

The stigma surrounding diabetes,^31^, ^35^, ^36^, ^37^, ^41^, ^42^, ^43^ insulin and injection^24^, ^25^, ^28^, ^29^, ^36^, ^45^ as well as medication taking in public^27^, ^28^, ^31^, ^35^, ^36^, ^37^, ^38^, ^42^ were major impediments to medication adherence. Diabetes was associated with getting old,^37^, ^42^ ill^37^ and weak.^37^ Using insulin and injections were negatively perceived as “being a junkie”^25^ and “illicit drug use”.^36^ Some PwT2D tried to conceal their diagnosis and not take their medications in public.^31^, ^35^ This was observed for both insulin^36^, ^38^ and oral medication.^37^

Other people's experience with diabetes and medications are elaborated in Section 3.2.5 on the interactions between COM‐B components.

#### 3.2.3 Motivation

Both reflective and automatic aspects of motivation were described by PwT2D as affecting their medication adherence.

##### Automatic motivation

Habit and routine,^24^, ^25^, ^26^, ^27^, ^31^, ^33^, ^34^, ^35^, ^37^, ^38^ fear of needles, injections and blood,^24^, ^25^, ^28^, ^29^, ^32^, ^35^, ^38^, ^39^, ^40^, ^41^, ^45^ negative feelings towards medications,^24^, ^25^, ^28^, ^29^, ^36^, ^37^, ^41^ burnout, and self‐care fatigue^24^, ^25^, ^31^, ^33^ were some factors related to automatic motivation.

Some PwT2D struggled to integrate medication taking into their life,^24^, ^25^, ^35^ while others cultivated the habit of taking their medications successfully.^25^, ^26^, ^27^, ^31^, ^33^, ^34^, ^35^, ^37^, ^38^ For example, they “developed the reflex” of always having their medications with them.^27^

Eleven studies reported that PwT2D's fear of needles, injections and blood deterred them from taking their medications.^24^, ^25^, ^28^, ^29^, ^32^, ^35^, ^38^, ^39^, ^40^, ^41^, ^45^ Some were afraid of self‐injection.^25^, ^32^, ^41^ Some were afraid because they had personally experienced pain from injections^29^, ^32^, ^40^ or associated injections with their previous unpleasant blood‐taking experiences.^41^ This resulted in delayed initiation^35^, ^38^, ^45^ and omission of medications.^38^, ^40^

Studies described several ways in which PwT2D's negative feelings towards medications^24^, ^25^, ^28^, ^29^, ^36^, ^37^, ^41^ could affect adherence. Insulin provoked negative emotions,^24^, ^36^ such as panic^36^ and anger.^41^ Some PwT2D blamed themselves and felt a sense of failure for requiring insulin^24^, ^25^, ^28^ and this was reinforced by the negative social influences, which are described in Section 3.2.2 on social opportunity. Some PwT2D felt sad for having to take medications daily^36^, ^37^ and said, for example, that they “really hate medicines”.^37^ Negative feelings and beliefs about medications may overlap and are described in Section 3.2.5 on the interactions between COM‐B components.

Burnout and self‐care fatigue hindered medication adherence.^24^, ^25^, ^31^, ^33^ Some PwT2D were frustrated over prolonged medication intake.^33^

##### Reflective motivation

The largest number of themes were related to reflective motivation. More than 10 studies discussed the perceived necessity and effectiveness of medications,^25^, ^26^, ^27^, ^28^, ^29^, ^30^, ^31^, ^32^, ^33^, ^35^, ^36^, ^37^, ^38^, ^39^, ^41^, ^42^, ^44^, ^45^ personal ownership,^24^, ^25^, ^26^, ^27^, ^28^, ^29^, ^30^, ^31^, ^33^, ^34^, ^35^, ^36^, ^37^, ^38^, ^44^, ^45^ concern about diabetes symptoms and complications,^24^, ^25^, ^26^, ^27^, ^29^, ^31^, ^33^, ^35^, ^36^, ^37^, ^38^, ^42^, ^43^, ^44^, ^45^ side effects of medications,^25^, ^26^, ^27^, ^28^, ^29^, ^30^, ^31^, ^32^, ^33^, ^35^, ^36^, ^37^, ^38^, ^39^, ^40^, ^41^, ^42^, ^43^, ^44^ greater value placed on competing commitments and priorities,^24^, ^25^, ^26^, ^27^, ^28^, ^33^, ^35^, ^38^, ^39^, ^40^, ^43^ and self‐adjustment of medication.^25^, ^30^, ^31^, ^32^, ^35^, ^36^, ^37^, ^40^, ^42^, ^43^, ^44^

Studies also reported that some PwT2D hope to avoid or stop insulin in the future.^25^, ^26^, ^27^, ^28^, ^35^, ^36^, ^38^, ^45^ Additionally, some studies described self‐efficacy,^25^, ^38^, ^39^, ^40^, ^41^, ^45^ fatalism,^24^, ^25^, ^31^, ^37^, ^41^, ^42^, ^44^ self‐identity and the concept of being sick,^24^, ^25^, ^27^, ^33^, ^35^, ^37^, ^41^, ^42^, ^44^ as well as the negative association of insulin with severe diabetes and the perception of insulin as the last resort.^24^, ^25^, ^28^, ^29^, ^36^, ^37^, ^41^ Some PwT2D's preferences for alternative medications^31^, ^35^, ^36^, ^37^, ^42^, ^44^ and nonpharmacological management of diabetes,^30^, ^35^, ^36^, ^43^ perceptions towards certain medications and brands,^29^, ^37^, ^42^ concerns about needle hygiene, device issues and source of insulin^28^, ^38^, ^41^ were also raised.

The perceived necessity and effectiveness of medications was a common factor influencing PwT2D's decision to adhere.^24^, ^25^, ^26^, ^27^, ^28^, ^29^, ^30^, ^31^, ^32^, ^33^, ^35^, ^36^, ^37^, ^38^, ^39^, ^41^, ^42^, ^44^, ^45^, ^46^ Some PwT2D found medications unnecessary^30^, ^36^ and felt fine without taking their medications^30^, ^33^, ^35^, ^37^, ^44^ whereas others believed that medications were necessary and effective, when they witnessed improvement in their diabetes control^27^, ^30^, ^32^, ^35^, ^38^, ^45^ and felt better physically with medication adherence.^26^, ^27^, ^29^, ^35^ These positive results reinforced their motivation to continue adhering to their medications.^38^

Personal ownership influenced medication adherence.^24^, ^25^, ^26^, ^27^, ^28^, ^29^, ^30^, ^31^, ^33^, ^34^, ^35^, ^36^, ^37^, ^38^, ^44^, ^45^ Some PwT2D understood the importance of medication adherence; however, they lacked the discipline to commit to it.^31^ Some PwT2D were motivated to take their medications if they had the desire to live.^31^, ^33^, ^36^, ^44^ Some acknowledged that it was their responsibility to take their medications^24^, ^25^, ^30^, ^35^, ^37^, ^46^ and devised their own strategies to ensure regular medication use.^26^, ^27^, ^31^, ^33^, ^34^, ^35^, ^40^, ^46^, ^47^

Eleven studies reported that PwT2D self‐adjusted their medications based on their food intake, lifestyle, glucose levels, side effects and symptoms.^25^, ^30^, ^31^, ^32^, ^35^, ^36^, ^37^, ^40^, ^42^, ^43^, ^44^ They increased their medication dose to make up for their missed dose^35^ and when they had dietary indiscretion.^36^, ^37^, ^43^ Some skipped their medications when they considered their blood sugar level as “okay”^31^ whereas others self‐adjusted their medications when they experienced side effects^25^, ^31^, ^32^, ^40^, ^43^ or based on their perception of their body cues.^42^, ^43^

Eight studies found that some PwT2D were motivated to take their medications in the hope of stopping or avoiding insulin in the future.^25^, ^26^, ^27^, ^28^, ^35^, ^36^, ^38^, ^45^ For example, some hoped that adhering to their oral medications now would prevent their future need for insulin^25^, ^26^, ^27^, ^36^, ^46^ and believed that they could stop insulin if their diabetes was well controlled in the future.^25^, ^35^, ^38^, ^45^

Self‐efficacy facilitated medication adherence.^25^, ^38^, ^39^, ^40^, ^41^, ^45^ When PwT2D saw that the insulin device was easy to use, they became more confident and receptive to the medications.^41^, ^45^ They were confident of taking their medications once they had adapted to them.^38^, ^41^

Self‐identity and the concept of being sick were also expounded in nine studies.^24^, ^25^, ^27^, ^33^, ^35^, ^37^, ^41^, ^42^, ^44^ Some PwT2D struggled to accept that they had diabetes^24^, ^25^, ^27^, ^33^, ^42^ as they did not have symptoms.^33^ One stated that “I didn't say that I have diabetes. I said THEY said I have diabetes”.^24^ Meanwhile, some felt like a different person because of their medications^25^ and struggled to accept their “new identity as an insulin user”.^24^ While some did not deny their diabetes, they downplayed its severity and considered diabetes “not a disease at all”.^42^ Some did not take their medication seriously due to the perceived minor consequences of not taking their medications.^35^, ^37^, ^44^

Having to take insulin was associated with having severe diabetes and the “last resort” by many.^24^, ^25^, ^28^, ^29^, ^36^, ^37^, ^41^ There was a belief that insulin was used when diabetes was severe^28^, ^29^ and could not be controlled by other medications.^25^, ^28^, ^29^, ^41^ They were resistant to taking insulin when they did not think that their diabetes was severe.^25^, ^28^, ^41^

Fatalism hindered medication adherence.^24^, ^25^, ^31^, ^37^, ^41^, ^42^, ^44^ This may be attributed to religious faith,^31^, ^37^, ^42^ the incurability and chronicity of diabetes,^24^, ^31^, ^44^ and the prolonged need for medications.^24^, ^35^, ^36^, ^37^, ^38^, ^44^ Some believed that only God can cure their diabetes^42^ and depended on divine interventions.^31^, ^37^ The incurability of diabetes discouraged some from taking their medications.^44^

There was a preference for using herbal, natural or alternative medications.^31^, ^35^, ^36^, ^37^, ^42^, ^44^ They were believed to be more effective,^35^, ^42^ safer^36^ and had fewer side effects^35^, ^36^, ^44^ than conventional medications.

There was also a preference for nonpharmacological approaches, such as dietary and lifestyle changes.^30^, ^35^, ^36^, ^43^ Several PwT2D believed that medication was not needed if they had a healthy diet and regular physical activity.^30^, ^35^

PwT2D's perceptions towards certain medications and brands^29^, ^37^, ^42^ seemed to affect their medication adherence too. Some PwT2D in Kuwait and India felt that overseas brands had greater efficacy and less side effects than local brands.^29^, ^42^ In contrast, others felt that local brands were “fresh”^42^ and distrusted overseas brands because they were perceived as “poisons”,^42^ “chemicals”^37^ and “toxic”.^37^

Concerns about side effects of medications, diabetes symptoms and complications, greater value placed on competing commitments and priorities are described in Section 3.2.5 on the interactions between COM‐B components.

#### 3.2.4 Personality and adherence

One study briefly proposed that PwT2D's personality, for example carelessness, may affect their medication adherence.^31^ This new theme could not be mapped onto the COM‐B model due to limited information.

#### 3.2.5 Interactions between COM‐B components

Although many adherence factors reported by patients could be mapped onto one specific COM‐B component, some could be mapped onto two components. Moreover, there were several ways in which they could interact with each other (Figure 3).

##### Adherence and motivation

The outcomes of PwT2D's adherence and motivation were linked in some studies.^24^, ^25^, ^30^, ^32^, ^36^, ^37^, ^42^ Some doubted the effectiveness of medications and were frustrated when their diabetes was poorly controlled despite medication adherence.^24^, ^30^, ^32^, ^36^, ^37^, ^42^ One said that “you try to do everything, but then it looks like it doesn't matter”.^24^

##### Capability and motivation

PwT2D's psychological capability also seemed to influence their reflective and automatic motivation, and vice versa.^25^, ^26^, ^28^, ^31^, ^32^, ^33^, ^34^, ^35^, ^36^, ^37^, ^38^, ^39^, ^40^, ^41^, ^42^, ^43^, ^44^, ^45^

The lack of understanding about diabetes and medication led some PwT2D to doubt the necessity and effectiveness of the medications. For example, some PwT2D refused medications due to superstition and unawareness of the underlying causes of diabetes.^43^ Some believed that the medication was ineffective when they developed complications shortly after their diagnosis.^37^ Some PwT2D were illiterate, had difficulty understanding diabetes and medications, hence they felt fearful and “helpless” about starting insulin.^41^ Conversely, PwT2D who understood their medications exhibited self‐efficacy and felt “capable of taking medication for diabetes”.^25^, ^26^ When PwT2D understood that diabetes is progressive and they may eventually require insulin, they appreciated the necessity of insulin.^28^

Moreover, PwT2Ds' reasons for forgetting their medication may be secondary to a lack of reflective motivation. Some PwT2D shared that they still forgot their medication despite having reminders from their family.^33^ A number of PwT2D were not motivated to bring their medications along when they ate out, as they found bringing medications out “very troublesome”, and as a result they forgot their medications.^38^ Some PwT2D who brought their medications with them forgot to take their medications too as they were “involved with work”.^26^

##### Reflective and automatic motivation

Some interactions between reflective and automatic motivation factors were observed in the studies.

Side effects of medications were a major concern^25^, ^26^, ^27^, ^28^, ^29^, ^30^, ^31^, ^32^, ^33^, ^35^, ^36^, ^37^, ^38^, ^39^, ^40^, ^41^, ^42^, ^43^, ^44^ and a source of fear.^28^, ^29^, ^33^, ^35^, ^36^, ^37^, ^41^, ^42^, ^44^ Some PwT2D were afraid of hypoglycaemia.^28^, ^29^, ^41^, ^42^Those who had personally experienced side effects of medications^25^, ^29^, ^30^, ^32^, ^36^, ^38^, ^39^, ^40^, ^43^, ^44^ described having “metformin moments (diarrhoea)”,^25^ feeling “really ill”,^30^ “trembling badly”^39^ when they took their medications. This overlap between their concern^28^, ^35^, ^38^, ^41^ and fear^24^, ^25^, ^28^, ^29^, ^32^, ^35^, ^38^, ^39^, ^40^, ^41^, ^45^ was also evident in their stance towards needles and injection.

Concerns about diabetes symptoms and complications were a dominant factor associated with medication adherence,^24^, ^25^, ^26^, ^27^, ^29^, ^31^, ^33^, ^35^, ^36^, ^37^, ^38^, ^42^, ^43^, ^44^, ^45^ often accompanied by fear.^24^, ^25^, ^26^, ^27^, ^33^, ^37^, ^38^, ^42^, ^44^ Some PwT2D took their medications to avoid developing diabetes complications.^24^, ^25^, ^26^, ^36^, ^37^, ^38^, ^42^, ^44^ PwT2D who had personally experienced diabetes symptoms^36^, ^38^, ^44^ and developed complications^27^, ^33^, ^37^, ^42^, ^44^ were more adherent to their medications, especially if their symptoms and complications were contributed by medication nonadherence.^25^, ^33^, ^37^, ^44^

Many PwT2D had the reflective motivation to plan their medication intake, which eventually became a habit and an automatic motivation for them.^25^, ^26^, ^27^, ^35^ For example, they placed their pillbox somewhere visible as a cue to take their medications.^35^

##### Opportunity and motivation

Moreover, other people's experience with diabetes^25^, ^26^, ^28^, ^33^, ^37^, ^38^, ^44^ and medications^25^, ^26^, ^28^, ^33^, ^37^, ^41^, ^44^ influence PwT2D's motivation and perception towards their diabetes and medications. Some PwT2D were more adherent to medications after learning about others experiencing poor diabetes control and its complications,^26^, ^28^, ^38^ due to reasons such as medication nonadherence.^26^ On the contrary, some PwT2D seemed nonadherent to medications if they knew others who passed away soon after starting medications,^37^ experienced social restrictions due to medications,^28^ lived long lives despite not adhering to medications^48^ or had poor diabetes control despite adhering to medications.^44^ Others' experience fuelled their fear of medications^25^, ^28^, ^37^, ^41^ and diabetes complications.^26^, ^38^

Competing commitments and priorities were often cited as reasons for medication nonadherence.^24^, ^25^, ^26^, ^27^, ^28^, ^33^, ^35^, ^38^, ^39^, ^40^, ^43^ PwT2D's competing commitments at work,^25^, ^26^, ^27^, ^38^, ^40^ family^25^, ^38^, ^43^ social activities^27^, ^28^, ^35^, ^38^, ^40^ were related to opportunity in the COM‐B model, as they were factors lying outside the PwT2D.^12^ Nevertheless, they were mapped onto reflective motivation because of the higher value PwT2D placed on these competing priorities that affected their medication adherence.

Some PwT2D felt that HCPs overprescribed insulin and “too many medications”.^42^ They reduced their medication intake because they were concerned that these medications would compromise their immunity.^42^ Notably, the HCP's explanation had also influenced PwT2D to perceive insulin as the last resort.^39^

Stigma surrounding diabetes and medications made some PwT2D felt guilty,^27^ embarrassed^27^ and ashamed.^37^ Furthermore, some were introduced to alternative medications by their family and friends^35^, ^37^ and opted for herbs, which were cheaper than conventional medications.^31^

##### Opportunity and capability

Opportunity factors appeared to influence PwT2D's capability too.

The lack of communication and poor relationship with HCPs^27^, ^29^, ^30^, ^31^, ^33^, ^34^, ^35^, ^37^, ^38^, ^39^, ^42^, ^43^, ^44^, ^45^ may have contributed to PwT2D's insufficient understanding about diabetes and medication.^25^, ^28^, ^29^, ^31^, ^33^, ^34^, ^35^, ^37^, ^41^, ^42^, ^43^, ^45^ Some PwT2D stated that they did not understand diabetes and its management, as HCPs prescribed and explained their medications very briefly^37^ and did not counsel them on their condition or medication.^35^, ^37^ By contrast, education and demonstration^28^, ^34^, ^35^, ^37^, ^39^, ^45^ by HCPs helped PwT2D to understand their medications,^45^ acquire skills^39^, ^45^ and develop self‐efficacy in medication taking.^39^, ^45^ This is an example of how opportunity factors affected capability factors, which in turn also affected their motivation.

Additionally, metformin is an example of a medication that is too large in size, challenging some PwT2D's physical swallowing capability, resulting in reluctance in taking it.^31^

##### Physical and social opportunity

Overcrowded clinics and short consultations potentially limited HCPs' communication with PwT2D.^37^ Some PwT2D described HCPs prescribing their medications in “two minutes”. It was difficult to discuss their concerns with the HCPs because of the short consultation.^27^

## 4 DISCUSSION

To the best of our knowledge, this is the first review of qualitative studies mapping different factors associated with medication nonadherence among PwT2D onto the COM‐B model. A narrative review of qualitative studies was published in 2014, but it did not map the factors onto a behavioural model and provided seven broad categories of barriers, such as *intentional nonadherence* and *medication administration*,^11^ in contrast to our more granular factors using the COM‐B model. Moreover, as 22 more studies have been published since then, our review ensures that the evidence is now up to date. Our hypothesized COM‐B variant model (Figure 3) captures various interactions between the capability, opportunity and motivation factors in an attempt to reflect the complexity of the different factors.

The largest number of themes identified were mapped onto motivation, particularly reflective motivation. Many studies elaborated PwT2D's perceived necessity and effectiveness of medications,^25^, ^26^, ^27^, ^28^, ^29^, ^30^, ^31^, ^32^, ^33^, ^35^, ^36^, ^37^, ^38^, ^39^, ^41^, ^42^, ^44^, ^45^ as well as concern about their side effects.^25^, ^26^, ^27^, ^28^, ^29^, ^30^, ^31^, ^32^, ^33^, ^35^, ^36^, ^37^, ^38^, ^39^, ^40^, ^41^, ^42^, ^43^, ^44^ These major factors align with the necessity‐concern framework, which described a positive correlation between perceived necessity and adherence, a negative correlation between concerns and adherence, and how medication beliefs explained more variance in adherence than clinical and sociodemographic factors.^49^ Our review reaffirms that medication beliefs are a strong determinant of adherence among PwT2D. Besides, the overlap between reflective and automatic motivation observed in our review resonates with the Plans, Responses, Impulses, Motives and Evaluations (PRIME) theory of motivation, which proposed that human behaviour is driven by the interplay of reflective thought, emotions and habits.^50^

Our review findings are consistent with earlier studies.^11^, ^51^, ^52^ Pound *et al*.'s synthesis of qualitative adherence studies posited that medications influenced people's self‐identity and were associated with stigma.^51^ Some PwT2D struggled to accept their new identity as someone with diabetes or the need to inject insulin, and were significantly affected by the social stigma they experienced.

Resistance to insulin was generally associated with the fear of needles, injections and social stigma in previous studies.^11^, ^52^ However, studies included in our review delved deeper and revealed that acceptance of insulin by PwT2D may also be related to their perception and acceptance of their diabetes severity. Knowing the potential underlying reasons for aversion to insulin may facilitate the implementation of novel adherence support interventions, such as the Acceptance and Commitment Therapy, a psychological intervention that has demonstrated effectiveness for improving diabetes control and self‐care among PwT2D in a meta‐analysis.^53^

Our hypothesized interaction that physical opportunity barriers, such as busy clinics and time constraints may have contributed to limited communications between HCPs and PwT2D concurs with the previous synthesis.^11^ The cost of medications and consumables frequently mentioned in the literature^11^, ^52^ is highly dependent on government healthcare policies.^52^ This underscores a need to investigate and address medication adherence at systemic levels too.

Personality is a relatively novel minor theme identified in our review and not mentioned in other studies.^11^, ^52^ Although conscientiousness and agreeableness in the 5‐factor model of personality may be positively associated with medication adherence,^54^ the role of personality as a modifiable and actionable target in medication adherence remains debatable.^55^, ^56^ Further research is warranted to ascertain its influence and practical implication on medication adherence.

### 4.1 Strengths and limitations

A major strength of our review is applying the COM‐B model to classify the various factors associated with medication adherence in diabetes. Although this model was not decided using BFFS authors' recommended strategy^21^ due to time and resource constraints, the COM‐B model is an appropriate *best fit* a priori model relevant to medication adherence, as proposed in an earlier study.^12^ Most of the themes generated from the factors could be mapped onto this model. The helpful overlaps with this model allowed for faster and organized synthesis from the studies, as compared to inductive methods.^22^ As the COM‐B model is a published model, the revisions made to this model that led to our hypothesized variant model for PwT2D are transparent and can be easily evaluated by comparing Figures 1 and 3.

Our review included studies of PwT2D on oral and/or injectable medications from different countries with varying cultures and socioeconomic status. Therefore, our findings would probably be relevant to a large population of PwT2D, although the relevance of some factors, such as financial barriers, may vary depending on the countries and healthcare systems, in which the studies were undertaken.

All included studies were deemed to have satisfactory quality as they reported key criteria adequately. It could be argued that assessing study quality based on reporting quality may be too simplistic. Nonetheless, there is no consensus on the best approach for assessing qualitative research for inclusion in systematic reviews.^57^ Reporting quality is plausibly a determinant of study quality, as a study can only be properly assessed if it is adequately reported.^23^

BFFS is a relatively new synthesis method. While its innovative elements such as merging framework and thematic analysis^21^ may require further evaluation, its advantage of engaging with theory but not being restricted by it^21^ justified its use. It harnesses the advantages of both framework and thematic analysis, does not force themes into the model and captures themes falling outside of the model inductively.^21^

Besides, it is challenging to ascertain whether the absence of interactions between the COM‐B components in our hypothesized model reflects a genuine absence of interaction, as the included studies may not have reported or investigated these interactions. Nonetheless, our hypothesized model attempts to capture prominent interactions observed from the included studies and serves as a good starting point for future refinement.

Some factors may not fit into a COM‐B component neatly but span multiple components. For example, forgetfulness, which was mapped onto psychological capability, may also be mapped onto reflective motivation, as studies have found that forgetting or poor planning may be influenced by someone's medication beliefs.^58^ This is not a study limitation. Instead, it informs the need to illustrate the intersection between the COM‐B components and the complexity of medication adherence in our hypothesized model. It also highlights the importance of targeting a factor with multiple approaches in planning interventions to improve adherence.

### 4.2 Practical implications and future study

Very few existing interventions explicitly used theory, analysed individuals' specific barriers and were tailored to address these barriers.^14^ Hence based on our review, intervention function and behaviour change techniques that are likely to target specific COM‐B components effectively can be selected for personalized intervention development.^17^ For example, reflective motivation may be targeted through feedback on behaviour.

Our hypothesized model may be developed into a programme theory^21^ describing the key components, mechanisms and context of an intervention,^59^ which can be further examined in future studies.

## 5 CONCLUSION

Our review generated a hypothesized COM‐B variant model that describes the factors and their potential interactions influencing medication adherence among PwT2D. This theoretically grounded synthesis may facilitate future intervention development by formulating a programme theory and identifying behaviour change techniques to address the identified factors.

## AUTHOR CONTRIBUTIONS

Vivien Teo: Conceptualization, data curation, formal analysis, investigation, methodology, project administration, writing—original draft, review and editing.

John Weinman and Kai Zhen Yap: Conceptualization, methodology, supervision, writing—review and editing.

## COMPETING INTERESTS

There are no competing interests to declare.

## Appendix 1 Search strategy

The following search strategy was used for Medline and was adapted for the other databases. Search terms are free text terms, unless otherwise stated. MESH: Medical subject heading (Medline medical index term); exp: exploded MeSH; *: truncation that searches for root word with any endings. Adj: adjacent

Diabetes

1 MESH: exp Diabetes Mellitus/dt [Drug Therapy]
2 Diabet*.ab, ti.
3 1 or 2

4 MeSH: exp Medication Adherence/
5 MeSH: exp treatment refusal/
6 MeSH: exp patient dropouts/
7 MeSH: exp Self Administration/
8 MeSH: exp Health Knowledge, Attitudes, Practice/
9 4 or 5 or 6 or 7 or 8
10 (drug* or medication* or medicine* or therap* or treatment* or regimen* or prescription*).ab, ti.
11 (adher* or nonadheren* or non‐adheren* or complian* or noncomplian* or non‐complian* or persist* or concordan* or refus* or empower* or belief* or believe*).ab, ti.
12 (10) adj3 (11)
13 9 or 10 or 11 or 12

14 3 and 13
15 Apply publication limit to 14 above: From 2014 onwards

Medication adherence

Combining both search concepts

## Appendix 2 Study characteristics

**Table:**

Study	Main aim	Methodology	Analysis approach	Participant	Medication type	Country	Overall quality assessment
Jannuzzi *et al*. 2014^23^	To identify and analyse salient beliefs—behavioural, normative, control and self‐efficacy related to adherence to oral antidiabetic agents	Semi‐structured interview; Theory of planned behaviour	Content analysis	17 PwT2D, mean age 59.8	Oral	Brazil	Adequately described
Widayanti *et al*. 2021^38^	To explore any practical issues that potentially affect medication‐taking behaviours	Semi‐structured interview; Conceptual model of Patients' Lived Experiences with Medicines (PLEM)	Content analysis with a deductive approach	51 PwT2D, unspecified mean age	Oral, injectable	Indonesia	Adequately described
Baghikar *et al*. 2019^27^	To explore barriers and facilitators to adherence among low‐income, urban Latinos	Semi‐structured interview; Social Ecological Model	Modified template approach where coding was guided by initial codebook, which was further amended as themes emerged	27 PwT2D, mean age 57	Oral, injectable	USA	Adequately described
Hassali *et al*. 2014^39^	To gain insight into PwT2D’s perspectives and beliefs about use of insulin, explore barriers to initiation of insulin	Semi‐structured interview; Phenomenological approach	Thematic content analysis	13 PwT2D, mean age 59.8	Oral	Malaysia	Adequately described
Patel *et al*. 2015^25^	To explore attitudes towards insulin acceptance	Semi‐structured interview; Necessity‐concerns framework	Grounded theory, constant comparison approach	18 PwT2D, unspecified mean age	Oral, injectable	UK	Adequately described
Liu *et al*. 2022^35^	To determine the impact of patients' beliefs about insulin on acceptance and adherence to insulin	Semi‐structured interview	Grounded theory; Constant comparison and synthesis	21 PwT2D, mean age 61	Injectable	Singapore	Adequately described
Mathew *et al*. 2022^36^	To explore the key aspects of patient–provider relationship that may be related to insulin acceptance	Semi‐structured interview	Grounded theory; Constant comparison and synthesis, Thematic analysis	21 PwT2D, median age 63	Injectable	Singapore	Adequately described
Bockwoldt *et al*. 2017^22^	To describe experiences of taking diabetes medications and identify factors that influence these experiences	Semi‐structured interview; Roy adaptation model	Thematic analysis	15 PwT2D, mean age 51.7	Oral, injectable	USA	Adequately described
Alhaddad *et al*. 2015^43^	To explore barriers to medication adherence	Semi‐structured interview	Framework analysis which involves the use of a thematic framework	20 PwT2D, mean age 53.7	Oral, injectable	Kuwait	Adequately described
Rezaei *et al*. 2019^37^	To explore the inhibitors of medication adherence in PwT2D	Semi‐structured interview	Conventional content analysis based on the method suggested by Lundman and Graneheim	12 PwT2D, mean age 52	Oral, injectable	Iran	Adequately described
Ahmad *et al*. 2021^32^	To investigate patients' medication‐taking behaviour and factors that influence adherence at the 3 phases of adherence	Semi‐structured interview	Thematic analysis	23 PwT2D, median age 39	Oral, injectable	Australia	Adequately described
Onwuchuluba *et al*. 2021^28^	To explore patients' experience of living with diabetes and taking their prescribed medication	Semi‐structured interview	Thematic analysis based on socioecological framework	25 PwT2D, unspecified mean age	Unspecified	Nigeria	Adequately described
Sapkota *et al*. 2016^33^	To explore Nepalese participants' perceptions of diabetes treatment	Semi‐structured interview	Thematic analysis, constant comparison approach	48 PwT2D, median age 55.5	Oral, injectable	Australia and Nepal	Adequately described
Jiraporncharoen *et al*. 2020^41^	To understand the relationship between patient's attitudes and medication adherence for oral anti‐diabetics.	Semi‐structured interview	Thematic analysis following the World Health Organization framework, line‐by‐line inductive analysis, textual analysis	24 PwT2D, mean age 62	Oral	Thailand	Adequately described
Polonsky *et al*. 2021^29^	To expand current understanding of patients' experiences, motivations and challenges relevant to their persistence with glucagon‐like peptide‐1 receptor agonist therapy	Semi‐structured interview	Thematic analysis	36 PwT2D, mean age 58.3	Injectable	USA	Adequately described
Alzubaidi *et al*. 2015^34^	To explore and compare medication‐taking experiences and associated issues	Semi‐structured individual interview and group interview	Thematic analysis, constant comparative method	100 PwT2D, mean age 57 for Arabic speaking, 60 for English speaking	Oral, injectable	Australia	Adequately described
Okazaki *et al*. 2022^40^	To report key factors that motivate reluctant people to initiate insulin	Qualitative phone interview	Steps for coding and theorization (SCAT)	6 PwT2D, mean age 60.5	Injectable	Japan	Adequately described
Habte *et al*. 2017^26^	To elicit medication‐related beliefs	In‐depth interview; Necessity‐concerns model	Not specified, guided by Horne's necessity‐concerns model	39 PwT2D, unspecified mean age	Oral, injectable	Central Ethiopia	Adequately described
Guenette *et al*. 2015^57^	To elicit patients' beliefs about taking their oral antidiabetic drugs	Focus group; Theory of planned behaviour	Content analysis using TPB as the theoretical framework	45 PwT2D, mean age 63.8	Oral	Canada	Adequately described
Shiyanbola *et al*. 2018^30^	To explore reasons for medication nonadherence and adherence	Focus group; Phenomenology approach	Content analysis	40 PwT2D, mean age 53	Oral, injectable	USA	Adequately described
Bockwoldt *et al*. 2016^21^	To describe the perception of insulin treatment	Focus group; Roy adaptation model	Thematic analysis, constant comparative method	13 PwT2D, mean age 52 for mid‐life group, 70 for older group	Oral, injectable	USA	Adequately described
Hsu *et al*. 2014^31^	To elicit organizational facilitators and barriers that contribute to people's medication adherence	Focus group	Nonspecified—draft code list, coded, compared and revised code list	45 PwT2D, mean age 69.3	Oral	USA	Adequately described

*Note*: PwT2D: People with type 2 diabetes. Examples of injectable medication are insulin and glucagon‐like peptide‐1 receptor agonists.
